# An Overview of Biotransformation and Toxicity of Diterpenes

**DOI:** 10.3390/molecules23061387

**Published:** 2018-06-08

**Authors:** Ingrid P. de Sousa, Maria V. Sousa Teixeira, Niege A. Jacometti Cardoso Furtado

**Affiliations:** Department of Pharmaceutical Sciences, School of Pharmaceutical Sciences of Ribeirão Preto, University of São Paulo, Av. do Café, s/n, Ribeirão Preto, São Paulo 14040903, Brazil; ingrid.sousa@usp.br (I.P.d.S.); valdeline_sousa@hotmail.com (M.V.S.T.)

**Keywords:** diterpenes, biotransformation, toxicity

## Abstract

Diterpenes have been identified as active compounds in several medicinal plants showing remarkable biological activities, and some isolated diterpenes are produced at commercial scale to be used as medicines, food additives, in the synthesis of fragrances, or in agriculture. There is great interest in developing methods to obtain derivatives of these compounds, and biotransformation processes are interesting tools for the structural modification of natural products with complex chemical structures. Biotransformation processes also have a crucial role in drug development and/or optimization. The understanding of the metabolic pathways for both phase I and II biotransformation of new drug candidates is mandatory for toxicity and efficacy evaluation and part of preclinical studies. This review presents an overview of biotransformation processes of diterpenes carried out by microorganisms, plant cell cultures, animal and human liver microsomes, and rats, chickens, and swine in vivo and highlights the main enzymatic reactions involved in these processes and the role of diterpenes that may be effectively exploited by other fields.

## 1. Introduction

Diterpenes are, by definition, C_20_ compounds based on four isoprene (C_5_H_8_) units and can be found in plants, fungi, bacteria, and animals in both terrestrial and marine environments [1,2,3,4,5].

The biochemically active isoprene units, isopentenyl diphosphate and dimethylallyl diphosphate, may be derived from the mevalonate and deoxyxylulose phosphate pathways. The mevalonate pathway is active in all higher eukaryotes and many bacteria, while the deoxyxylulose phosphate pathway is operative in bacteria and in the chloroplasts of green algae and higher plants [6]. Both pathways are the natural routes for the synthesis of terpenoids and many other natural products that contain terpenoid elements in their molecules, in combination with carbon skeletons derived from the acetate and shikimate pathways [7].

Diterpenes seem to be biosynthesized predominantly by the deoxyxylulose phosphate pathway [4,7], but some authors reported that the mevalonate pathway can also supply different portions of a molecule, since there is exchange of late-stage common intermediates of both pathways supplying the isoprene units [7,8]. 

**Figure 1:**
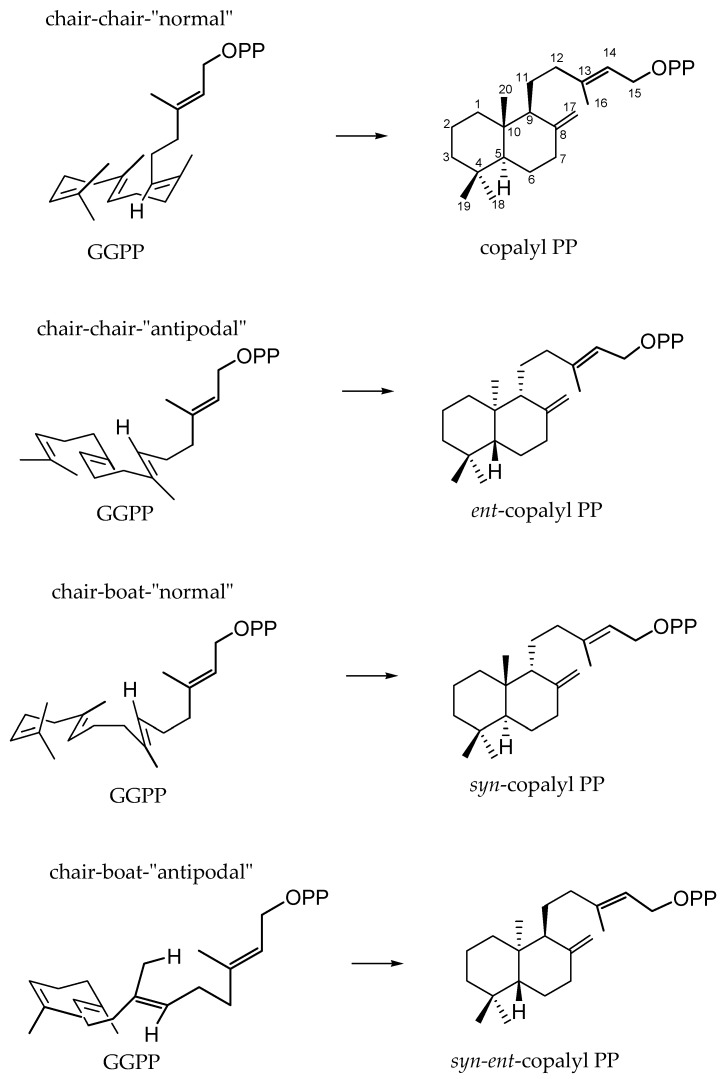
Conformational folds of geranylgeranyl diphosphate (GGPP) to copalyl diphosphate stereoisomers [10].

**Figure 2:**
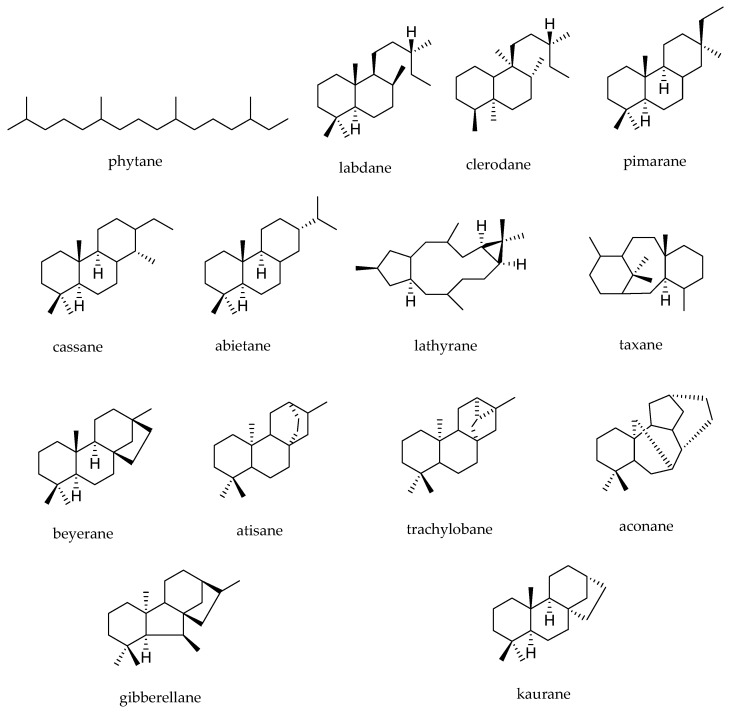
Common diterpene skeletal types.

The diterpenes arise from geranylgeranyl diphosphate (GGPP), which is formed by addition of isopentenyl diphosphate to farnesyl diphosphate [7]. The cyclisation of GGPP to diterpenes proceeds via four different stereochemical courses, starting from four different conformational folds: GGPP in the chair-chair-“normal” conformation leads to copalyl diphosphate; GGPP in the chair-chair-“antipodal” conformation leads to *ent*-copalyl diphosphate; GGPP in the chair-boat-“normal” conformation yields *syn*-copalyl diphosphate and GGPP in the chair-boat-“antipodal” conformation yields *syn*-*ent*-copalyl diphosphate [9] (Figure 1). Compounds that belong to the “normal” series are those whose fusion between A and B rings occurs in the same way as in steroids, while compounds of the series “ent” present structures that are specular images of the normal series [10]. The chair-boat conformation leads to “syn” orientation of the hydride and methyl substituents across the C-9-C-10 bond, which can occur with the “normal” or “ent” absolute configuration [10]. The range of diterpenes is extended from labdane-related diterpenes, which are the most basic hydrocarbon backbones, by cyclisation reactions leading to the production of bicyclic, tricyclic, tetracyclic, pentacyclic, and macrocyclic types, as well as diterpene dimers [3,10]. The most common diterpene skeletal types are shown in Figure 2. 

Diterpenes have been identified as active compounds in several medicinal plants showing remarkable biological activities [4,11,12,13,14]. Paclitaxel and ingenol-3-angelate are examples of important anti-cancer drugs [4] and forskolin, salvinorin A, triptolide, carnosic acid and ginkgolide B are prominent compounds showing cardioprotective, analgesic, anti-inflammatory, antioxidant and inhibitor of platelet activating-factor effects, respectively [4]. 

Diterpenes from different sources have also been shown cytotoxicity against various cancer cell lines, nitric oxide inhibitory activity, plant-growth regulating properties, phytotoxic activity on root growth, as well as antiplasmodial, hypoglycemic, hypolipidemic, antimicrobial, antiviral, antifouling, larvicidal, algicidal, and insect antifeedant activities, among others [2,3,15,16,17,18]. 

Because of the promising biological activities of diterpenes, there is great interest in developing methods to obtain derivatives of these compounds, and biotransformation processes are interesting tools for the structural modification of natural products with complex chemical structures, which are difficult to achieve using chemical reactions [19,20]. 

Biotransformations are useful not only to obtain a variety of new chemical structures and active compounds [21] but are also used to investigate metabolic pathways [20,22,23]. Microorganisms, for example, are used as predictive models for mammalian drug metabolism [24], and these organisms can also contribute with useful information in the search for new antimicrobial drugs by providing understanding of how microorganisms metabolize xenobiotics.

This review is intended to present an overview of biotransformation processes of diterpenes carried out by microorganisms, plant cell cultures, animal and human liver microsomes, and rats, chickens, and swine in vivo and highlights the main enzymatic reactions involved in these processes. Major emphasis was placed on recent publications reporting a variety of enzymatic reactions and a diversity of chemical structures, as well as on the available literature data regarding the biological activities of some biotransformation products. As diterpenes are produced by some organisms as a defense strategy against predators, parasites, and mechanical injury, this review covers literature data summarizing the antifeedant, insecticidal, and antifungal activities of diterpenes, which can be useful in the development of agrochemicals for crop protection products. 

## 2. Biotransformation of Diterpenes

According to IUPAC, biotransformation can be defined as the chemical conversion of substances by living organisms or enzyme preparations [25]. The term metabolism covers a broader definition of the entire physical and chemical processes of catabolism and anabolism to maintain life [25]. However, in medicinal chemistry, IUPAC defines metabolism as “biotransformation of xenobiotics and particularly drugs.” Some authors consider the biotransformation as one of the steps of drug metabolism, which includes absorption, distribution, biotransformation, and excretion [26]. For other authors, the drug metabolism comprises only the biotransformation step and consequent chemical modifications of drugs by the enzymes, and the absorption, distribution, metabolism (biotransformation), and excretion together constitute the pharmacokinetics [27]. Another definition for biotransformation is “the reaction of chemical compounds in a living system, and it need not be a process defined by the organism’s metabolism” [28]. This definition is in agreement with Hanson [29], who states that biotransformation is the use of biological systems to promote chemical transformations in non-natural substrates. In other words, biotransformation could be considered as the enzymatic modifications of xenobiotics and metabolism as the enzymatic modifications of endogenous compounds. Although there are different concepts for metabolism and biotransformation, it is common to see authors using both terms with the same meaning in chemistry of natural products. It seems that the terms biotransformation, metabolism, bioconversion, and biocatalysis are interchangeable and often used as synonyms in the literature. To the best of our knowledge, no definitive agreement has been established so far, but the definition proposed by Hanson [29] seems to be more appropriate.

Human biotransformation of xenobiotics has classical steps classified in two phases (phase I and phase II) to inactivate and convert hydrophobic compounds into more hydrophilic derivatives [30]. The biotransformation facilitates metabolite excretion through the urine or the bile and avoid toxic effects from the accumulation of non-polar xenobiotics in the body. However, more toxic, reactive, or carcinogenic metabolites can be produced during the biotransformation process [30]. 

The drug metabolizing enzymes of phase I carry out oxidation, reduction and hydrolysis reactions and consist mainly of the cytochrome P450 (CYP) enzyme superfamily and flavin-containing monooxygenases. The phase II enzymes are mainly transferases, which catalyze conjugation of the substrate with polar endogenous groups such as sulfate (sulfotransferases), glucuronic acid (UDP-glucuronosyltransferases), glycine (glycine *N*-acyltransferases), glutamine (glutamine *N*-acyltransferases), glutathione (glutathione *S*-transferases), acetyl (acetyl-transferases) and methyl (methyl-transferases) [27]. Xenobiotics can also undergo conjugation reactions with endogenous fatty acid and cholesterol [27]. These metabolizing enzymes can be found in the intracellular membranes and in the cytosol of the cells [30].

In the human body, biotransformations occur mostly in the liver and gastrointestinal tract, but also in tissues of the nasal and ocular mucosa, lung, kidneys and brain [30,31].

Not only the human metabolizing enzymes can modify the chemical structures of xenobiotic compounds, but also the enzymes from the human microbiota. For instance, the gut microbiota is a complex system that can act on dietary components, environmental pollutants and pharmaceuticals and modify their bioactivity, bioavailability and toxicity in a different way occurring in the human cells, since many enzymes are exclusively microbial [32]. 

Therefore, biotransformation has a crucial role in the process of a drug development and/or optimization. The understanding of the metabolic pathways for both phase I and II biotransformation of new drug candidates is mandatory for toxicity and efficacy evaluation and part of preclinical studies [33].

Some biocatalysts models to study biotransformation in vitro include microorganisms, such as bacteria, filamentous fungi, yeasts, plant cells, and microalgae [34]. Furthermore, intestinal and liver models such as immortalized cell lines (e.g., Caco-2 and HT-29) [35], intestinal organoids [35], isolated perfused liver [33], enzyme preparations (e.g., liver microsomes, cytosolic and S9 fractions) [33], membrane biohybrid systems of human hepatocytes [36], primary human hepatocytes with membrane bioreactors [36], and liver-on-chip models [37] have been also used.

It is possible to study biotransformation reactions in animal [38] or human [39] in vivo by collecting metabolites in the plasma, bile, urine, or feces. Despite the greater resemblance between the in vivo model and the true in vivo situation, there are demanding regulatory, safety, and ethical issues to be considered [33]. Furthermore, in the in vivo model, it is generally not possible to isolate metabolites for further toxicological evaluation, what is a great advantage of the microbial biocatalysts. In this context, filamentous fungi are a classical model for in vitro biotransformation due to their ability to mimic metabolic routes of xenobiotics in mammals [22] and the possibility of greater yields for metabolite isolation and evaluation. A more modern approach of fungal metabolic engineering, which combines the use of these microorganisms with molecular genetic tools and computer science, can expand the role of fungi not only in the bioproduction [40] but also in the biotransformation studies.

Another possibility to study biotransformation is using in silico methods through which is possible to computationally predict the metabolism of drugs based on chemical data sets of molecules, enzymatic metabolic reaction system and ligand- and structure-based design principles [41].

Biotransformations are useful not only to investigate metabolic pathways, but are also used to obtain a variety of new chemical structures and active compounds. Diterpenes can undergo several enzymatic reactions. The most common ones are the introduction of hydroxyl groups catalyzed by the cytochrome P450 enzymes. Phase I enzymes can also promote reduction, hydrolysis and epoxidation of terpenoid substrates [42,43]. Reduction of carboxyl groups at non-active sites are important enzymatic reactions catalyzed by microorganisms, for example. Literature has shown that phase II enzymes such as sulfotransferases, UDP-glucuronosyltransferases, glycine *N*-acyltransferases and methyl-transferases can react with different diterpenes, increasing their molecular weight and polarity [44,45,46].

Diterpenes are a promising class for biotransformations because they display a wide range of biological activities. Most of the studies with biotransformation of diterpenes aim to obtain new active derivatives. Although diterpenes are common and widespread in natural medicines for oral use in folk medicine [47,48], a lower number of publications focus on the use of biotransformation to investigate their metabolism for safety assessment.

**Figure 3:**
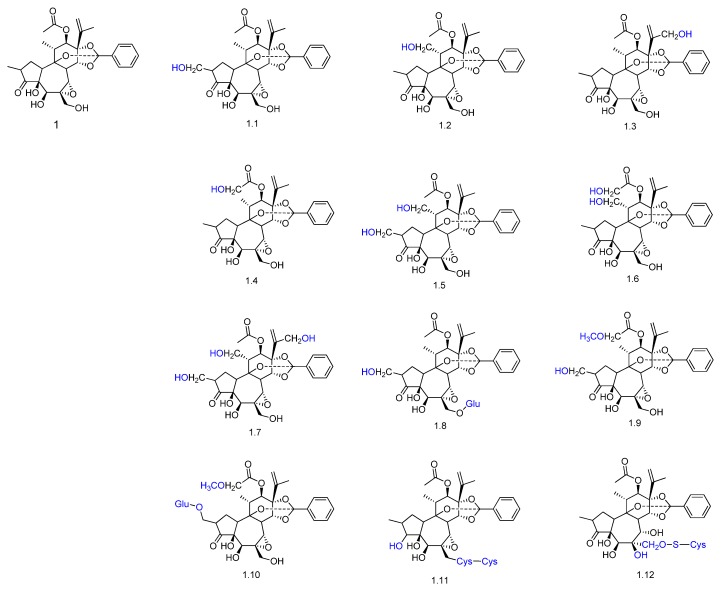
Proposed metabolites of yuanhuapine (**1**) detected in rat urine after oral administration [49].

Yuanhuapine (compound **1** in Figure 3) is a bioactive daphnane diterpenoid found in the plant *Daphne genkwa*, which is used in folk medicine for the treatment of edema, asthma, and cancer [49]. The metabolism of yuanhuapine was evaluated in rats, and 12 metabolites were identified by UPLC-Q-TOF/MS in rat urine after oral administration. Hydroxylation was the main enzymatic reaction observed in phase I metabolism (compounds **1.1**–**1.9**, Figure 3). Reduction was also observed (compound **1.11**). Metabolites conjugated with glucuronic acid (compounds **1.8** and **1.10**), cysteine (compounds **1.11** and **1.12**) and methylation (compounds **1.9** and **1.10**, Figure 2) were detected in phase II metabolism.

**Figure 4:**
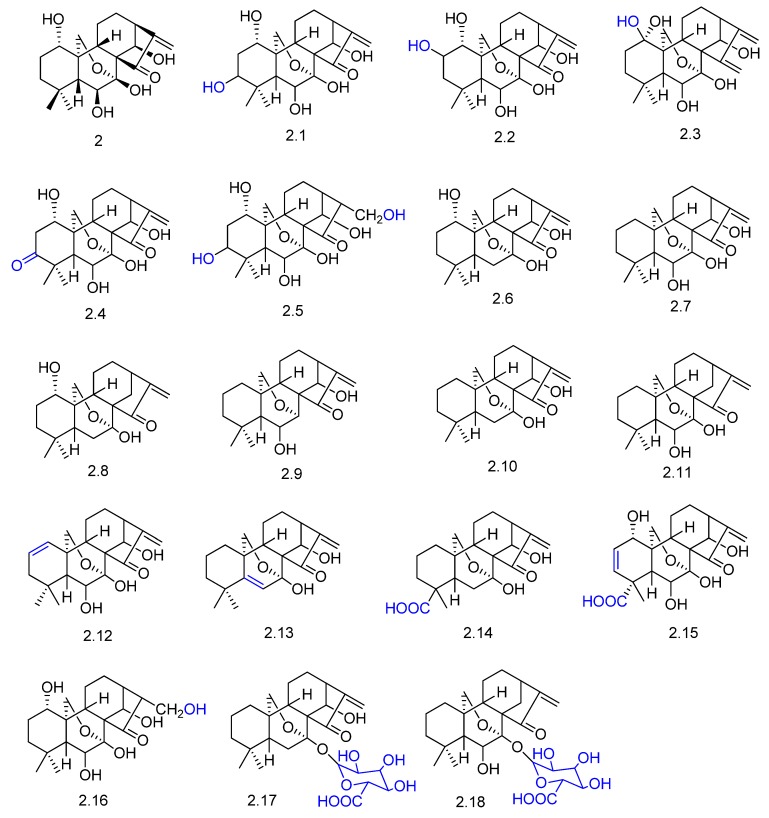
Proposed chemical structures of oridonin (**2**) metabolites in rats in vivo [50].

Oridonin (compound **2** in Figure 4) is an *ent*-kaurane diterpenoid and one of the major constituents of the Chinese herb medicine *Isodon rubescens* [50]. Driven by the need to better understand the metabolism of this active diterpene in vivo, oridonin was orally administrated in rats, and its metabolites were analyzed in bile and urine samples. The authors proposed 16 phase I metabolites (compounds **2.1**–**2.16**, Figure 4) originated mainly via hydroxylation, ketone formation, and dehydration. The conjugation of the phase I metabolites **2.10** and **2.11** with glucuronic acid originated two phase II metabolites, **2.17** and **2.18**, respectively (Figure 4).

**Figure 5:**
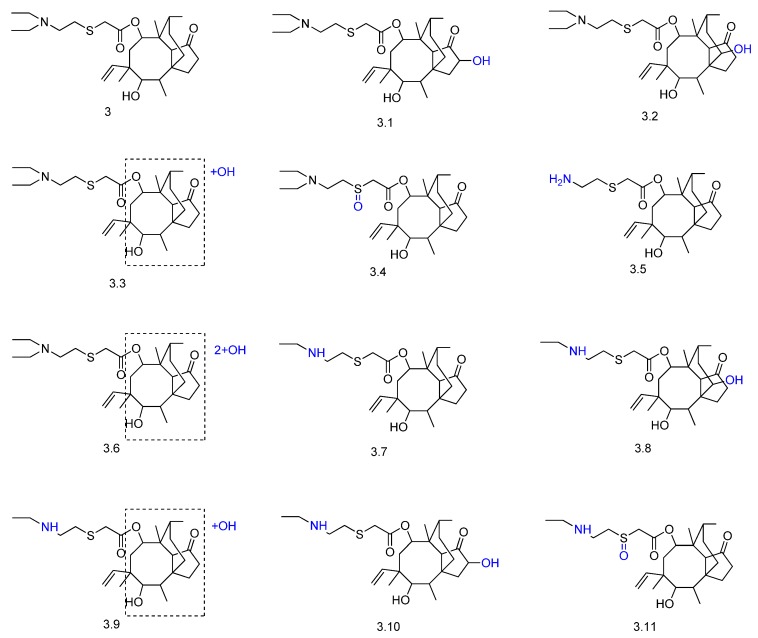
Proposed biotransformation metabolites of tiamulin (**3**) obtained in animals in vivo and in rats, swine, chickens, cows and goat liver microsomes [38].

Tiamulin (compound **3** in Figure 5) is a semisynthetic diterpene produced by fungi. It displays antimicrobial activity and has veterinary indications to treat dysentery and pneumonia in animals [38]. Tiamulin metabolism was investigated with rats, swine, chickens, cows, and goat liver microsomes and in rats, chickens, and swine in vivo. A total of 26 metabolites of phase I were identified by UHPLC-Q/TOF (compounds **3.1**–**3.11**, Figure 5). The main metabolic reactions in all animals were hydroxylation in the ring system, *S*-oxidation and *N*-deethylation on the side chain. No phase II metabolite was detected. The metabolism rate of tiamulin was greater in vivo than in microsomes, and qualitative and quantitative interspecies differences were reported.

**Figure 6:**
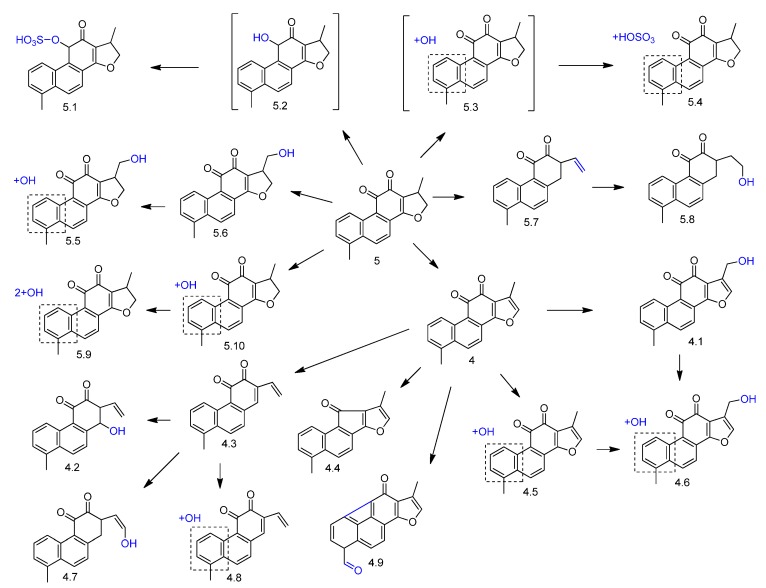
Proposed phase I and phase II metabolites from rat bile biotransformation of tanshinone I (**4**) and dihydrotanshinone I (**5**) [45].

Tanshinone I (compound **4** in Figure 6) is an abietane-type diterpene quinone found in *Salvia miltiorrhiza.* The metabolism of this compound has been investigated by different biotransformation approaches. According to Liu et al. [51], who studied the metabolism of a mixture of tanshinones (including compound **4**) using liquid chromatography/tandem mass spectrometry, oxidation was the dominant reaction after in vitro incubation with rat liver microsomes. The in vivo metabolism of **4** was also studied by the same authors after oral administration in rats and *O*-glucuronidation was proposed as the major pathway in phase II metabolism [51]. The rat bile biotransformation of **4** was also investigated together with its analogue dihydrotanshinone I (compound **5** in Figure 6) [45]. Fifteen metabolites of phase I were identified in the bile samples (compounds **5.5**–**5.10** and **4.1**–**4.9**, Figure 6). Hydroxylation, furan ring cleavage, oxidation, and dehydrogenation were among the main phase I reactions. The conjugation of dihydrotanshinone I (**5**) and the phase I metabolite **5.3** with *O*-sulfate afforded the phase II metabolites **5.1** and **5.4** (Figure 6).

**Figure 7:**
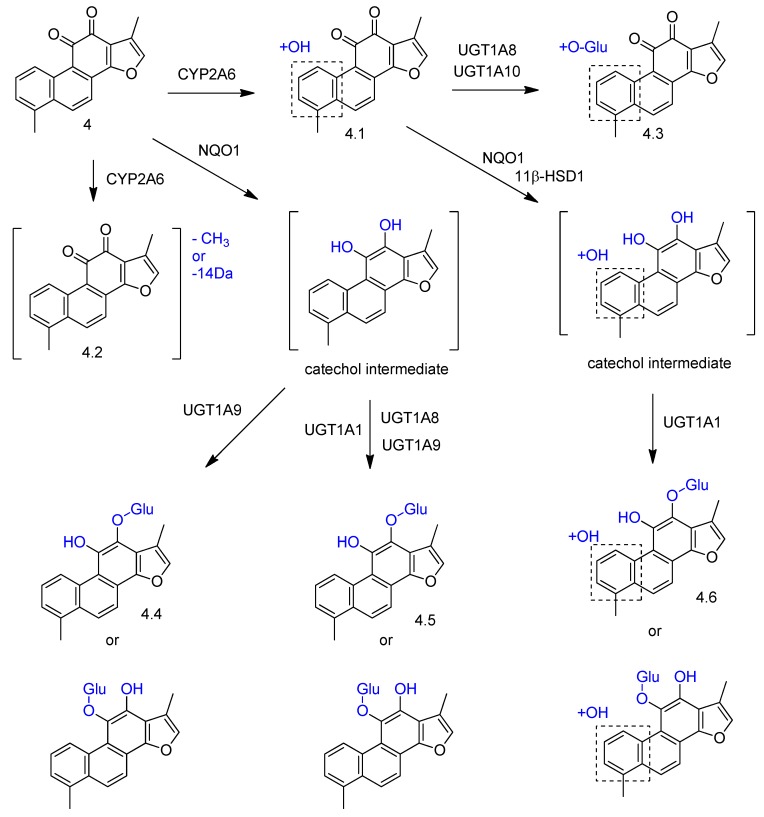
Proposed metabolic pathways and enzyme isoforms involved in the biotransformation of tanshinone I (**4**) by human liver microsomes and S9 subcellular fractions [52].

The biotransformation of tanshinone I with human liver microsomes and S9 subcellular fractions afforded similar metabolites than the ones described in the different biotransformation approaches, by hydroxylation, reduction, and glucuronidation enzymatic reactions (Figure 7) [52]. The investigation of the enzymatic isoforms involved in the bioconversions showed that CYP2A6 was primarily responsible for phase I metabolites (compounds **4.1** and **4.2**, Figure 7), and UGT1A1, UGT1A3, UGT1A7, UGT1A9, UGT1A8, and UGT1A10 were involved in phase II metabolism (compounds **4.3**–**4.6**, Figure 7). Tanshinone I (**4**) and compound **4.1** (Figure 7) were also reduced by NQO1 and generated catechol intermediates, which were immediately conjugated with glucuronic acid by the UGTs.

**Figure 8:**
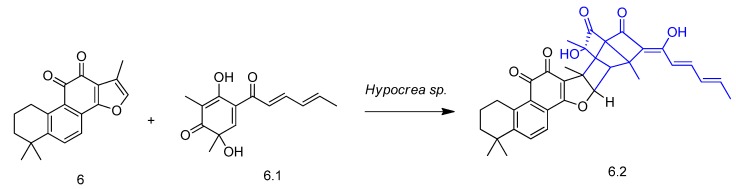
Bioconversion of tanshinone IIA (**6**) in tanshisorbicin (**6.2**) by *Hypocrea* sp. [53].

The biotransformation of tanshinone IIA (compound **6** in Figure 8), which displays a similar structure to tanshinone I (**4**) and was also isolated from *Salvia miltiorrhiza,* was investigated by He et al. [53]. The incubation of the diterpene with the fungus *Hypocrea* sp. afforded a new metabolite (tanshisorbicin, compound **6.2** in Figure 8) by [4 + 2] cycloaddition with sorbicillinol (compound **6.1**, Figure 8), which is a secondary metabolite produced by the fungus (Figure 8). For the first time, an enzymatic Diels-Alder type reaction with a secondary metabolite was reported in biotransformation studies. 

**Figure 9:**
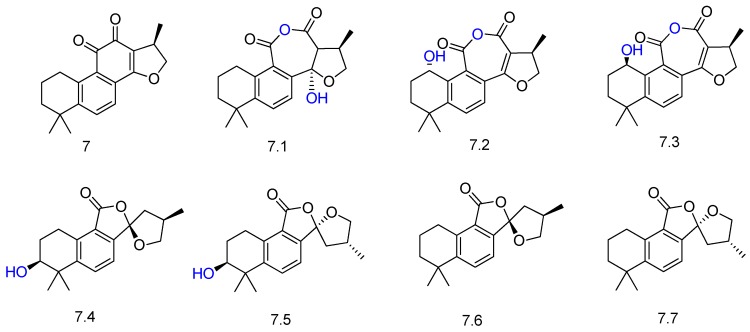
Derivatives **7.1**–**7.7** of cryptotanshinone (**7**) obtained by biotransformation with *Mucor rouxii* [54].

Another related diterpene quinone (cryptotanshinone, compound **7** in Figure 9) was submitted to biotransformation with *Mucor rouxii* and afforded seven ring-contracted derivatives (compounds **7.1**–**7.7**, Figure 9) [54]. The authors proposed the formation of the metabolites via conversion of the *ortho*-naphthoquinone to anhydride and rearrangement of **7** by the microbial enzymes. Among the biotransformation metabolites, **7.1**–**7.5** were described for the first time.

**Figure 10:**
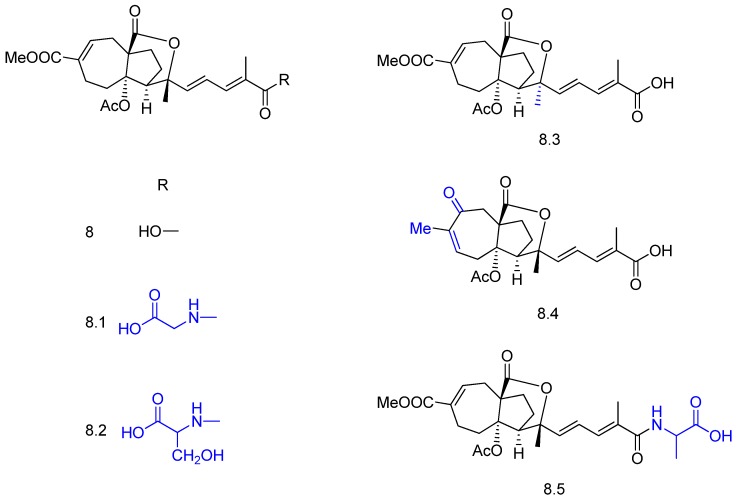
Derivatives (**8.1**–**8.5**) of pseudolaric acid (**8**) obtained by *Chaetomium globosum* biotransformation [46].

Pseudolaric acid B (compound **8** in Figure 10) is a diterpene that displays anticancer, antifertility and potent antifungal activities. The bioconversion of **8** by *Chaetomium globosum* afforded the three novel compounds pseudolaric acid I (**8.3**), pseudolaric acid B 18-oyl-alanine (**8.5**), and pseudolaric acid B 18-oyl-serine (**8.2**) [46]. Two known compounds, pseudolaric acid F (**8.4**) and pseudolaric acid B 18-oyl-glycine (**8.1**)m were also isolated from the biotransformation extracts (Figure 10). The bioconversions included conjugation with amino acids, epimerization, and migration of the double bond.

**Figure 11:**
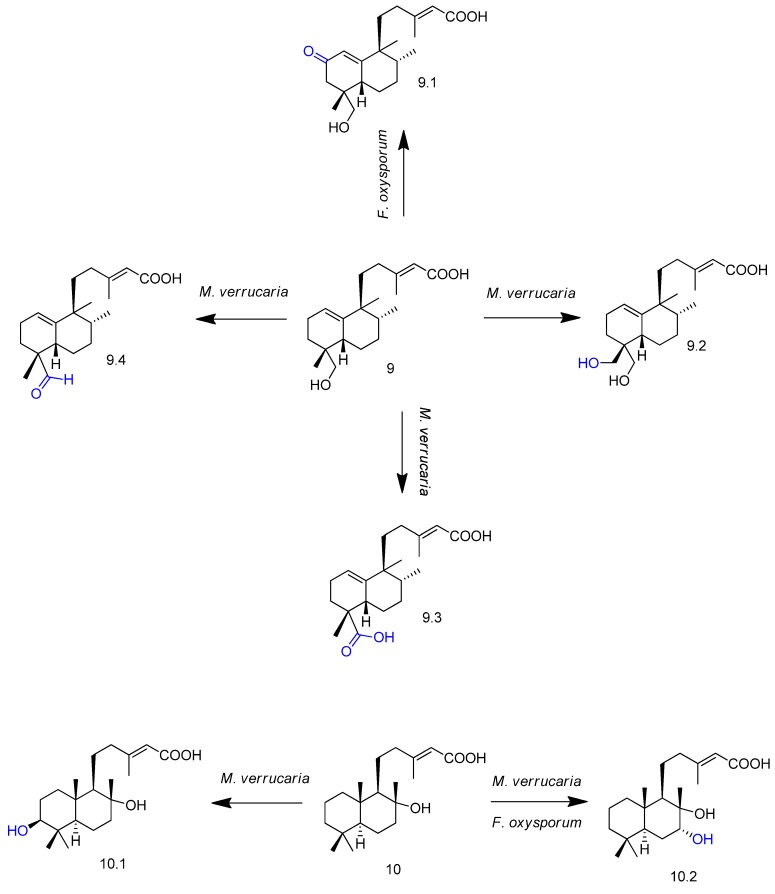
Diterpenoids **9** and **10** and their derivatives obtained by *Fusarium oxysporum* and *Myrothecium verrucaria* biotransformation [55].

The microbial transformation of the halimane diterpene (+)-(4*R*,5*S*,8*R*,9*S*)-18-hydroxy-*ent*-halima-1(10),13-(*E*)-dien-15-oic acid (compound **9** in Figure 11) and the labdane (+)-(5*S*,8*S*,9*R*,10*S*)-lab-13-en-8β-ol-15-oic acid (compound **10** in Figure 11) by *Fusarium oxysporum* and *Myrothecium verrucaria* afforded hydroxyl, oxo, formyl, and carboxy derivatives (compounds **9.1**–**9.4**, **10.1** and **10.2**, Figure 11) [55]. *F. oxysporum* showed preference to modify ring B from substrate **10**, while *M. verrucaria* was able to transform both rings A and B. Among the oxidized derivatives, compounds **9.1**, **9.2**, **9.4**, and **10.2** were reported for the first time.

**Figure 12:**
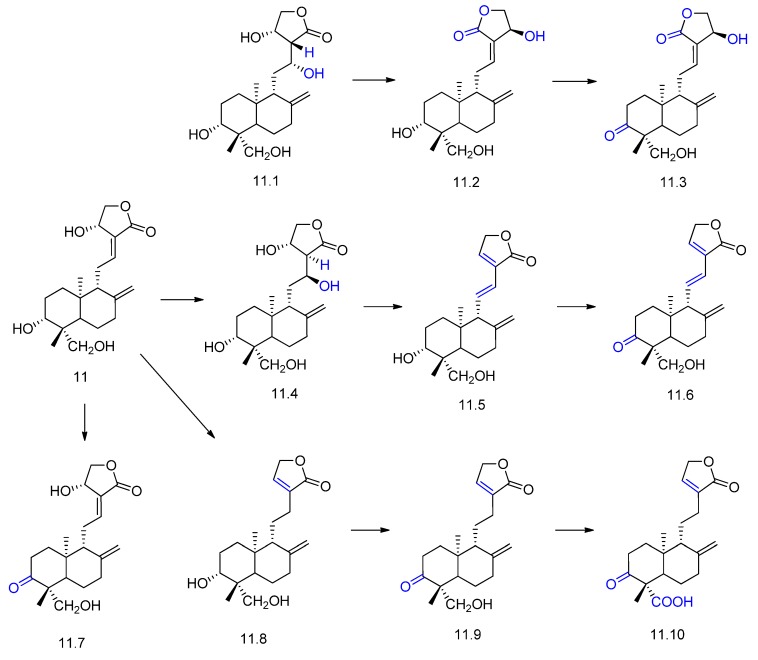
Andrographolide (**11**) and its derivatives **11.1**–**11.10** obtained by *Rhizopus stolonifer* biotransformation [57].

Andrographolide (compound **11** in Figure 12) is one of the main constituents of *Andrographis paniculata*, which is used in traditional Chinese medicine for the treatment of gastric disorders, infectious diseases, and common colds [56,57]. Andrographolide exhibits anti-inflammatory, immunomodulatory, hepatoprotective, anti-HIV, and antitumor effects. Its incubation with *Rhizopus stolonifer* yielded two novel biotransformation products (compounds **11.3** and **11.10**, Figure 12) and eight compounds (**11.1**, **11.2**, **11.4**–**11.0**, Figure 12) [57]. The main enzymatic reactions were oxidation and dehydration. The bioconversion of andrographolide with *Aspergillus ochraceus* afforded similar metabolites [56].

**Figure 13:**
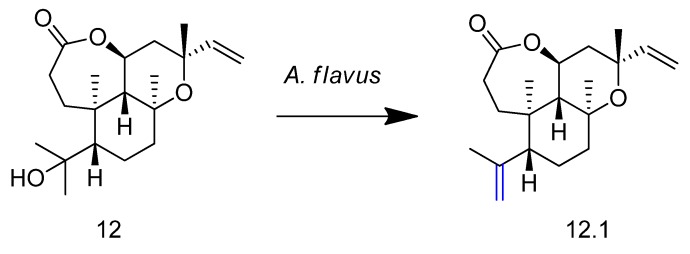
Biotransformation of agallochaexcoerin A (**12**) in agallochaexcoerin G (**12.1**) by *Aspergillus flavus* [58].

Sura et al. [58] studied the biotransformation of agallochaexcoerin A (compound **12** in Figure 13). The incubation of **12** with the pathogenic fungus *Aspergillus flavus* afforded the novel agallochaexcoerin G (compound **12.1**, Figure 13) via microbial biodehydration.

**Figure 14:**
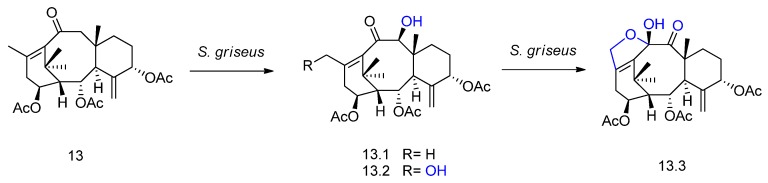
Biotransformation of compound **13** by *Streptomyces griseus* and its derivatives **13.1**, **13.2**, and **13.3** [59].

A derivative (compound **13** in Figure 14) obtained by chemical synthesis of the natural taxane sinenxan A was incubated with different microorganisms [59]. The bacterium *Streptomyces griseus* was selected for preparative scale biotransformation. Three new metabolites were isolated and identified, including two hydroxylated products (**13.1** and **13.2**) and a furantaxane with an unusual 6/8/6/5 ring system formed via oxidation of **13.2** and intramolecular acetalization (**13.3**, Figure 13).

**Figure 15:**
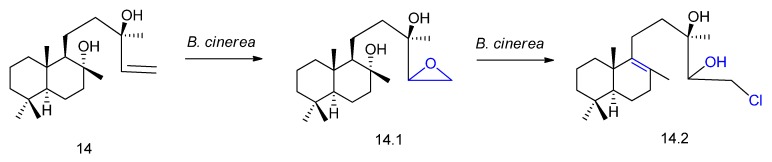
Epoxidation (**14.1**) and halogenation (**14.2**) of sclareol (**14**) by *Botrytis cinerea* [60]*.*

A rare example of diterpene halogenation was reported by Farooq and Tahara [60] using the fungus *Botrytis cinerea.* The microorganism transformed sclareol (compound **14** in Figure 15) in epoxysclareol (**14.1**), followed by bioconversion in the novel halogenated metabolite **14.2** (Figure 15). The authors suggest an epoxide opening via C-8,9-dehydration to afford a 14,15-dihydroxy intermediate followed by nucleophilic displacement of the hydroxyl group by a chlorine atom, mediated by the fungal enzyme. 

The biotransformation of sclareol was extensively studied with different types of microorganisms including *Cunninghamella echinulata*, *Cunninghamella elegans*, *Mucor plumbeus*, *Aspergillus alliaceus*, *Aspergillus niger*, *Aspergillus ochraceus*, *Curvularia lunata*, *Mortierella ramanniana*, *Mortierella isabellina*, *Rhizopus arrhizus*, *Rhizopus stolonifer* and *Sporotrichum exile* [61], the marine derived fungi *Xylaria* sp., *Botryosphaeria* sp. and *Eutypella* sp. [62], and the fungus *Botrytis cinerea* [63]. The most common metabolites are the hydroxylated derivatives 3β-hydroxysclareol, 18-hydroxysclareol, and 6α-hydroxysclareol.

**Figure 16:**
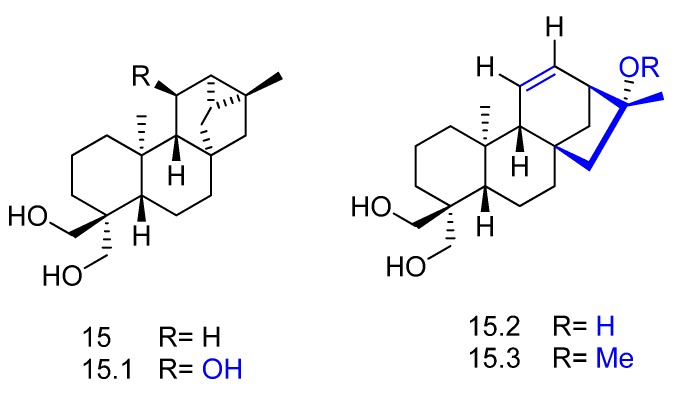
Chemical structures of **15** and its derivatives **15.1**, **15.2** and **15.3** obtained by biotransformation with *Rhizopus stolonifer* [64]*.*

The fungus *Rhizopus stolonifer* was used in the biotransformation of the diterpene *ent*-18,19-dihydroxytrachylobane (compound **15** in Figure 16) and yielded the new *ent*-11β,18,19-trihydroxytrachylobane (**15.1**) via C-11 hydroxylation of precursor **15** [64]. The biotransformation of **15**, which is an *ent*-trachyloban type diterpene, also yielded the new *ent*-kaurene type diterpenes *ent*-16α,18,19-trihydroxykaur-11-ene (**15.2**) and *ent*-18,19-dihydroxy-16α-methoxykaur-11-ene (**15.3**) (Figure 16). Metabolites **15.2** and **15.3** were probably resulted from a backbone rearrangement of **15.1**.

**Figure 17:**
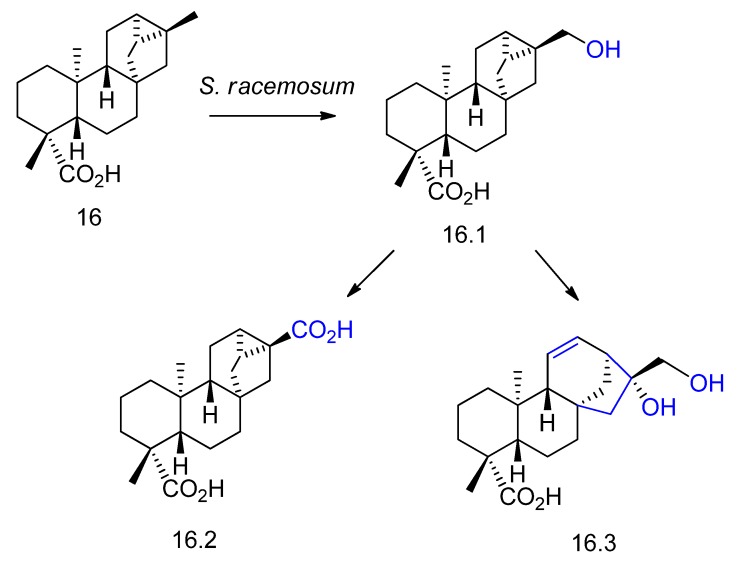
Chemical structures of the derivatives **16.1**, **16.2**, and **16.3** obtained by biotransformation of trachyloban-19-oic acid (**16)** with *Syncephalastrum racemosum* [65]*.*

Trachyloban-19-oic acid (compound **16** in Figure 17), a related structure to compound **15**, was incubated with *Syncephalastrum racemosum* in order to obtain new bioactive compounds [65]. Hydroxylation of **16** afforded the compound **16.1**, and oxidation of **16.1** yielded the new compound **16.2** (Figure 17). Another new metabolite was obtained by rearrangement of **16.1** into a kaurane hydroxylated diterpene (**16.3**, Figure 17).

**Figure 18:**
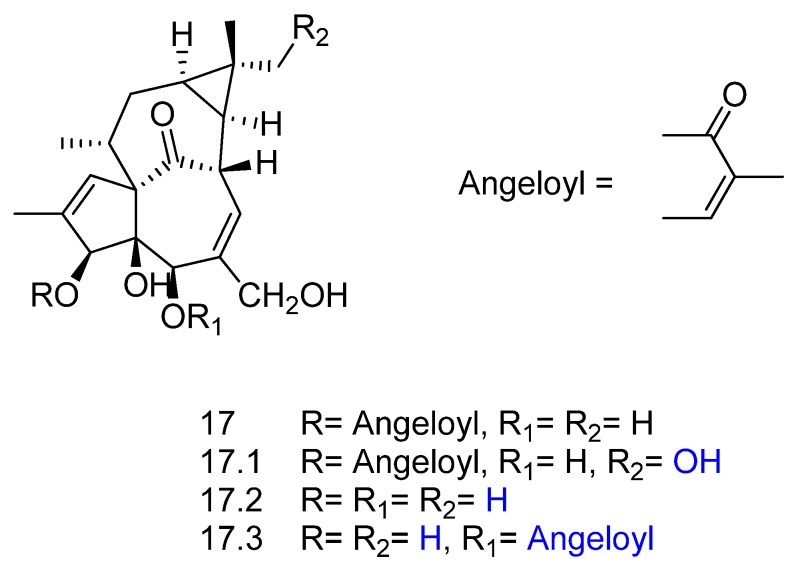
Chemical structures of ingenol-3-angelate (**17**) and its derivatives **17.1**, **17.2**, and **17.3** obtained by biotransformation with plant cell cultures [66]*.*

Ingenol-3-angelate (compound **17** in Figure 18) is a diterpenoid ester with noticeable anticancer and antileukemic activity. Teng et al. [66] studied its biotransformation by four different plant cell suspension cultures, namely *Hordeum vulgare*, *Oryza sativa*, *Panax quinquefolium*, and *Nicotiana tabacum*. Three biotransformation metabolites were detected in the culture medium of the plant cell suspensions (compounds **17.1**–**17.3**, Figure 18). The metabolite 16-hydroxy-ingenol-3-angelate (**17.1**) was produced via hydroxylation of the methyl group at C-16. A deacylated product originated the metabolite ingenol (**17.2**), and an acyl rearrangement of **17** afforded ingenol-5-angelate (**17.3**).

**Figure 19:**
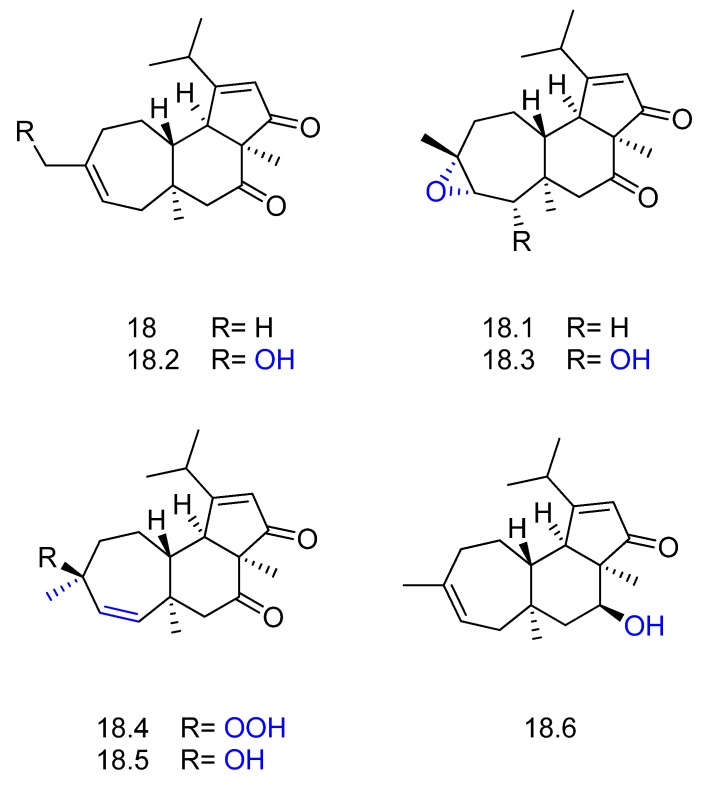
Structures of cyanthwigin B (**18**) and its derivatives **18.1**–**18.6** obtained by biotransformation with actinomycete *Streptomyces* sp. [67]*.*

In the search for active metabolites, Peng et al. [67] carried out the biotransformation of the 5,6,7-tricarbocyclic diterpene cyanthwigin B (compound **18** in Figure 19) with several microorganisms. The actinomycete bacteria *Streptomyces* NRRL5690 and *Streptomyces spheroids* were selected for preparative-scale biotransformation. After 5–10 days of incubation of the substrate with the microorganisms, six metabolites were isolated and identified, including the new compounds **18.1**, **18.2** and **18.3** and the known compounds **18.4**, **18.5**, and **18.6** (Figure 19). The main enzymatic reactions involved epoxidation, hydroxylation, and reduction.

Biotransformation has an important role in drug discovery. Biocatalysis can modify non-activated carbons of the molecule, which is interesting for the chemodiversity and obtainment of new chemical structures [43,44,61,68]. Diterpenes in general have few reactive sites for chemical modifications by semi-synthesis [69]. However, the literature data shows that they have good susceptibility toward biocatalysts enzymes. The biotransformation examples reported in the present review show that different carbons of the diterpene skeleton are susceptible to be modified by biotransformation. 

More recently, the possibility of combination of whole-cell biotransformations with artificial metalloenzymes, which catalyze new-to-nature reactions, is a potential source of new chemical molecules and can contribute in the drug discovery as new biocatalysts with a large repertoire of enzymatic reactions [70].

Biotransformation studies are useful not only to obtain a variety of new chemical structures and active compounds but also to understand how microorganisms metabolize xenobiotics, providing useful information in the search for new drugs against certain microorganism. The drug metabolism and the biotransformation processes performed by *Mycobacterium tuberculosis* have been combined with metabolomics-based studies and/or genetic strategies to better understand possible metabolic activation or inactivation of drugs [71]. The use of these combined strategies can be the most effective way to develop new antitubercular agents. 

Another important contribution of biotransformation studies for new drugs discovery is to enable increasing functionalization of the substrate and produce oxygenated derivatives with improved biological activity. 

In the search for new bioactive natural compounds, Cano et al. [72] studied the biotransformation of sclareolide by eight different species of filamentous fungi. The incubation with *Aspergillus niger* ATCC 16404, *Cunninghamella blakesleeana* ATCC 8688a, *Rhizopus nigricans* ATCC 6227b, and *Fusarium moniliforme* ATCC 10209 afforded the new product 3α,6β-dihydroxysclareolide. The biotransformation product displayed activity against a greater variety of cancer cell lines than the parent compound, showing that the hydroxyl groups at the C-3α and C-6β positions enhanced the biological activity of sclareolide. 

The *ent*-2*α*-hydroxylation of the *ent*-kaurane diterpene *ent*-15*α*-hydroxy-kaur-16-en-19-oic acid by *Fusarium proliferatum* produced a metabolite with higher allelopathic activity on the germination and growth of *Lactuca sativa* [73]. 

Other study showed that the microbial C-3α hydroxylation of the pimarane-type diterpene *ent*-8(14),15-pimaradiene increased its antimicrobial activity against multidrug-resistant bacteria [74]. Interestingly, the hydroxylation of C-3 combined with other hydroxyl or carbonyl groups in different positions of the pimarane skeleton decreased the antimicrobial potential of the metabolite. 

Sepúlveda et al. [75] studied the gastroprotective effects of derivatives obtained by both *Mucor plumbeus* biotransformation and chemical synthesis of the mulinane diterpene mulin-11,13-dien-20 oic acid combined with β-cyclodextrin. The authors obtained six new chemical structures by chemical synthesis, but the most active compounds were those obtained by biotransformation. These metabolites displayed greater gastroprotective effect than the parent compound and presented similar effects as the reference drug lansoprazole. One of the hydroxylated metabolites was even more active than the reference drug. This study suggests that the increase in the gastroprotective activity was related to the presence of the hydroxyl group, since the activity was decreased when this group was acylated.

Among 14 oxygenated compounds obtained by microbial biotransformation of isostevic acid, 12 of them displayed more potent inhibitory activity in the expression of the COX-2 mRNA than the control dexamethasone [76]. The enzymatic degradation and rearrangement of cryptotanshinone performed by *Mucor rouxii* originated two metabolites with enhanced activity against influenza A virus [54].

Twenty-four analogues were obtained by biotransformation of deoxyandrographolide with *Cunninghamella blakesleeana* [77]. Among them, four displayed significant inhibitory effect against LPS-induced NO production in RAW 264.7 macrophages. The bioconversion of the 3-OH group of the parent compound into a 3-ketone group was associated with the greater biological activity. The authors also associated a non-hydroxyl group at C-3 with the increase of the inhibitory effect. 

The biotransformation of andrographolide with *Aspergillus ochraceus* was investigated by He et al. [56], and five products were isolated. Among them, a metabolite produced by a sequence of hydration, dehydration, and oxidation enzymatic reactions exhibited the best cytotoxicity activity against human breast cancer MCF-7 and human colon cancer HCT-116 cell lines. This metabolite showed better activity than andrographolide and the positive control cisplatin. On the other hand, the potential of this derivative against leukemia HL-60 cell line was not enhanced when compared to the parent compound. The biological activities of andrographolide derivatives obtained with *Rhizopus stolonifer* were also investigated by He et al. [57]. Ten biotransformation products were obtained, and isoandrographolide displayed the best antiproliferative activity against MCF-7 and HCT-116 cell lines, being more active than the parent compound. Interestingly, only for the cell line HL-60 the metabolite isoandrographolide displayed weaker activity than its precursor. 

The bioconversion of andrographolide was also investigated with isolated enzymes. Its enzymatic transgalactosylation catalyzed by β-galactosidase from bovine liver afforded 19-*O*-β-galactosyl andrographolide [78]. This glycosylated product displayed antibacterial activity against food-borne pathogenic bacteria that were not affected by the precursor andrographolide [78]. Some 14-acylated derivatives synthetized by immobilized *Candida antarctica* lipase B showed antiproliferative effects against Gram-positive and Gram-negative bacteria [79]. Andrographolide exhibited no antimicrobial activity, whereas 14-butyrylandrographolide displayed the strongest antibacterial effect. The chain length of the acyl moiety on the lactone-ring of the derivatives displayed a critical role in determining the antibacterial activity.

The hydroxylation and dihydroxylation carried out by *Streptomyces griseus* on the substrate 10-oxo-2*R*,5*R*,14β-triacetox-ytaxa-4(20),11(12)-diene resulted in no significant effects on the tumor MDR reversal activity when compared to the parent compound [59]. Nevertheless, the enzymatic oxidation followed by acetalization of the dihydroxylated metabolite afforded a furantaxane derivative with potent reversal activity in the A549/taxol MDR tumor cell line. The reversal fold of the furantaxane derivative was two times higher than that of the parent compound and comparable with the positive control verapamil.

Driven by the need to discover new drug leads for the treatment of Alzheimer’s disease, Dos Santos et al. [65] screened the acetylcholinesterase (AChE) inhibitory activity of derivatives of trachyloban-19-oic acid obtained by biotransformation with *Syncephalastrum racemosum.* The enzymatic oxidation and rearrangement of the parent compound afforded three metabolites with improved biological activity. A carboxylic acid derivative displayed the best anti-AChE activity and was shown to be six times more active than the positive control galanthamine.

As reported herein, there are several examples of biotransformations affording more active compounds. On the other hand, it is also possible that some chemical modifications result in no significant effect on the biological activity or even decrease it.

Leverrier et al. [80] evaluated the cytotoxicity of six new hydroxylated metabolites derived from *ent*-trachyloban-18-oic acid biotransformation with *Rhizopus arrhizus*. All the biotransformation products displayed lower activity against KB and HCT-116 cancer cell lines when compared to the starting material. 

The hydroxylation promoted by fungi at the aliphatic acid ester side chain of the ingenane diterpene 13-oxyingenol dodecanoate decreased its cytotoxic effects against human colon cancer cell line Caco-2, breast cancer cell line MCF-7 and Adriamycin (ADM)-resistant cell line MCF-7/ADM. Four hydroxylated derivatives of 20-deoxyingenol, as well as their precursor, displayed no biological activity against the same cell lines [81].

The detoxification process, common to human metabolism of xenobiotics, can also occur in microbial cells. For instance, when incubated with the potent antifungal pseudolaric acid B, *Chaetomium globosum* bioconverted the diterpenoid into five inactive metabolites [46]. The fungi stopped growing in the presence of pseudolaric acid B, but after two days of incubation, the mycelia continued growing.

Despite the variability of effects on the biological activity, biotransformations can be an effective approach to enhance the activity of diterpenes for different biological effects, as reported in Table 1.

Furthermore, biotransformations can promote regio and stereoselective or regio and stereospecific reactions, leading to compounds that would be difficult, more expensive, or impossible to obtain by traditional organic synthesis [63,83]. 

A screening using marine-derived fungi as biocatalysists showed that *Eutypella* sp. and *Botryosphaeria* sp. were able to selectively hydroxylate the C-3 position of different terpenoids in the equatorial orientation [62].

Enzymes from plant cell cultures were able to selectively hydroxylate only one methyl group of the four methyl groups available in the diterpene ingenol-3-angelate, which can be considered a challenging reaction to obtain by chemical synthesis [66].

The strain *Fusarium proliferatum* has shown the ability to promote a selective hydroxylation at the C-2 position of *ent*-kaurane diterpenoids, with high biotransformation yields [73]. 

*Mortierella ramanniana* was able to perform the regio-selective hydroxylation at C-16 and C-19 and the stereo-selective hydroxylation with α-orientation at C-12 of the ingenane diterpenoid 20-deoxyingenol [81]. 

Porto et al. [74] reported the stereoselective hydroxylation by *Aspergillus ochraceus* at positions C-3, C-7 and C-11 of *ent*-8(14),15-pimaradiene, which would be very difficult to achieve by classical synthesis. 

*Mucor plumbeus* has shown preference for equatorial hydroxylation of the C-3 position from trachylobane-type diterpenes [84]. The clerodane diterpene 3,12-dioxo-15,16-epoxy-4-hydroxycleroda-13(16),14-diene was incubated with *Cunninghamella echinulata* and *Rhizopus stolonifer* and both fungi produced the *ent*-neo-clerodane diterpene (3*R*,4*S*,5*S*,8*S*,9*R*,10*S*)-3,4-dihydroxy-15,16-epoxy-12-oxo-cleroda-13(16),14-diene as a single product [85]. 

The diterpene lactone andrographolide was bioconverted by immobilized *Burkholderia cepacia* lipase and the product 14-acetylandrographolide was formed exclusively by regioselective acylation [86].

The 72 h incubation of grandiflorenic acid with *Fusarium graminearum* was able to almost completely convert the *ent*-kaurane diterpene grandiflorenic acid into the single 12α-hydroxygrandiflorenic metabolite [87]. 

These examples show the applicability of biotransformations for selective modifications of diterpenes and structurally related molecules. One of the great advantages of selective modifications is the need of fewer purification steps to obtain metabolites of interest.

The potential to produce new biologically active and enantiomerically pure compounds under mild conditions with stable enzymes may reflect the growing number of patents with biotransformation processes registered during the years. For diterpenes, there are patents using biotransformation approaches to prepare plant growth regulators, sweeteners and fragrance materials, antiproliferative, anticancer and immunosuppressive agents [68]. These patents highlight the importance of diterpenes biotransformation in the global industry.

## 3. Toxicity of Diterpenes

Although diterpenes show remarkable biological activities, some may be toxic for humans, resulting in acute or chronic impacts on different tissues and organs. A common side effect of paclitaxel chemotherapy, for example, is the development of peripheral neurotoxicity [88]. In addition, the risk of severity of adverse effects can be increased when paclitaxel is combined with other drugs [89]. The diterpene ingenol-3-angelate is the most recent drug that has been approved to treat actinic keratosis, and the most common side effects of this compound are local skin reactions such as erythema, flaking/scaling, and crusting [90]. Nevertheless, the evaluation of the benefit–risk balance of these diterpenes is favorable.

Besides the approved drugs paclitaxel and ingenol-3-angelate, other diterpenes from different sources have shown toxic effects in human and livestock. Diterpenes from *Euphorbia pekinensis*, for example, cause irritation of the skin, oral, and gastrointestinal mucosa in humans [91], as well as nephrotoxicity when in long term use or taking in great quantity [92]. Livestock may be poisoned by feeding on species of Euphorbiaceae (spurge) that contain irritant diterpene ester toxins [93], and their milk cause intoxication and even death when consumed by children [93]. These diterpenes may also act as tumor promoters as a result of direct or indirect exposure through consumption of animal products containing toxic compounds [94].

**Figure 20:**
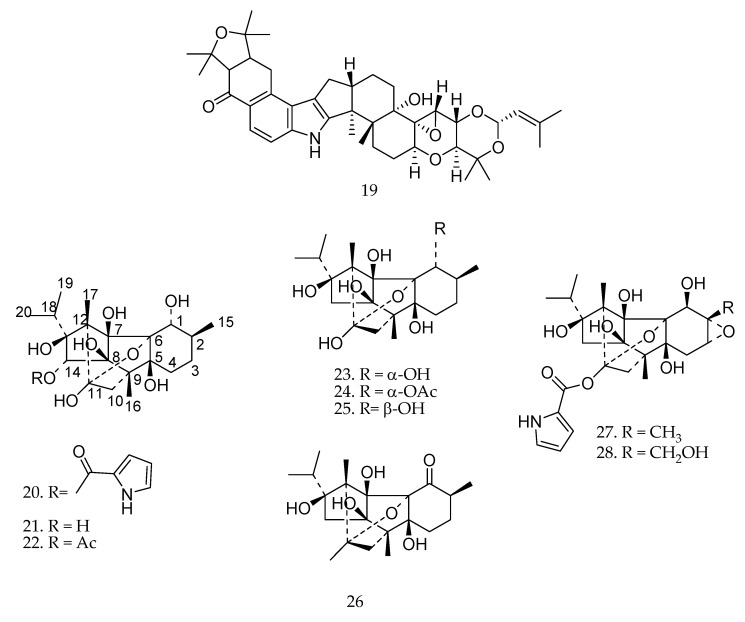
Chemical structures of diterpenes **19**–**28** [100,101].

Another toxic diterpene is lolitrem B (compound **19** in Figure 20), an indole-diterpene alkaloid produced in *Lolium perenne* (perennial ryegrass) infected by *Epichloë festucae* var. *lolii*, responsible for “ryegrass staggers” in livestock [95]. Affected animals develop uncontrollable tremors and become uncoordinated in their movement [96].

Diterpenes are produced by some organisms as a defense strategy against predators, parasites and mechanical injury and these compounds may be effectively exploited by other fields as, for example, in agriculture. Therefore, diterpenes can be useful in the development of insect antifeedants, insecticides, and antifungal agrochemicals for crop protection products [97,98]. 

In the 1940s, as a result of collaboration between Rutgers University and Merck in USA, a diterpene alkaloid extracted from *Ryania speciosa* (ryanodine, compound **20** in Figure 20) was discovered [99]. The diterpene alkaloid is moderately toxic to mammals if ingested, and residual activity of ryanodine has been reported for over one week after application [99].

The antifeedant activity of ryanodine against *Spodoptera litura* was also evaluated by Gonzaléz-Coloma et al. [100] together with six other diterpenes: ryanodol (**21**), ryanodol 14-monoacetate (**22**), ryanodine (**20**), cinnzeylanol (**23**), cinnzeylanine (**24**), epi-cinnzeylanone (**25**), and cinnzeylanone (**26**) (Figure 20). Compound **20** showed best antifeedant activity (*EC*_50_ = 1.220 ppm), followed by **25** (*EC*_50_ = 1.891 ppm), **23** (*EC*_50_ = 1.934 ppm), **21** (*EC*_50_ = 2.120 ppm), and **26** (*EC*_50_ = 2.598 ppm). Azadirachtin was used as positive control (*EC*_50_ = 0.247 ppm) [23]. Compounds **22** and **20** did not show significant activity (>3.0 ppm). 

A comparative study of antifeedant and insecticidal activities of ryanodol-type (compounds **21**–**26**) and ryanodine-type (compounds **20**, **27**, and **28**) diterpenes was carried out aiming to know the effects on feeding behavior, survivorship and performance of *Spodoptera littoralis* (larvae) and *Leptinotarsa decemlineata* (adults). The activity varied according to the treatment and the insect species. The β-stereochemistry at the C-1 position in **6** versus **3** and the *O*-acetylation at the compound **5** increased the toxic effect. Hydroxylation (**21**), *O*-acetylation (**22**), and pyrrolcarboxylate-esterification (**20**) at the C-14 position along the hydrophobicity of the cyclohexane ring resulted in intermediate activities. In addition, the presence of the epoxy-group (**27**, **28**) increased the toxicity (Figure 20). The presence of the ketone group (**26**) resulted in strong antifeedant activity [100,101].

**Figure 21:**
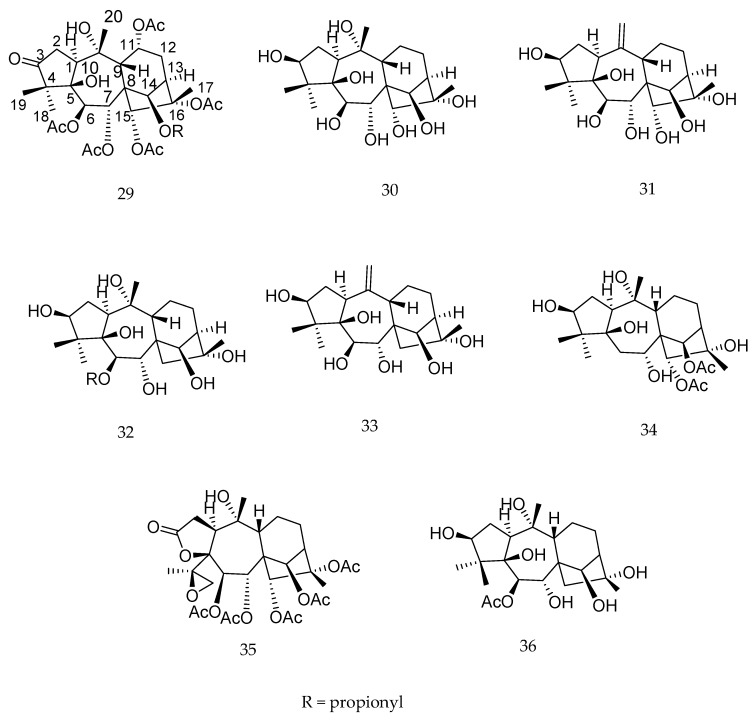
Chemical structures of diterpenes **29**–**36** [98,102].

Diterpenes from the genus *Pieris* have been highlighted as sources of insect antifeedant compounds [98]. The compounds pierisformosoid A (**29**), D (**30**) and I (**31**), asebotoxin (**32**) and kalmitoxin II (**33**) (Figure 21) at a dose of 0.5 mg/mL showed antifeedant activities against *Plutella xylostella*, with inhibition ratios of 92.3%, 60.2%, 73.7%, 78.8 and 45.7%, respectively [102]. The diterpenes pierisoid C (**34**) and E (**35**), together with asebotoxin IV (**36**) (Figure 21) exhibited antifeedant activity against the beet armyworm *Spodoptera exigua* with EC_50_ values of 10.91, 33.89, 6.58 µg·cm^−2^, respectively [98]. 

**Figure 22:**
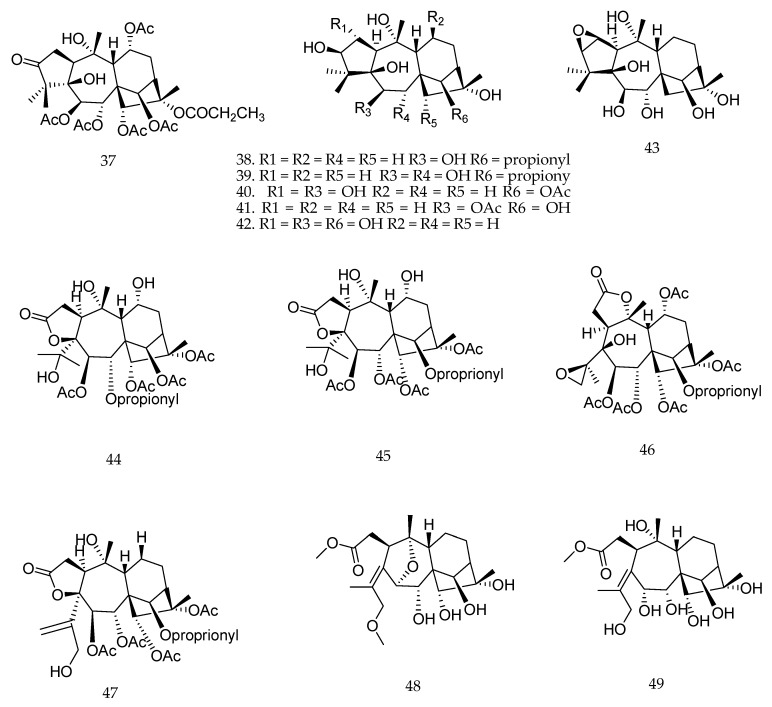
Chemical structures of diterpenes **37**–**49** [103].

Chen et al. [103] reported that grayanane diterpenoids (**37**–**43**) (Figure 22) showed potent antifeedant activity against *Pieris brassicae* (EC_50_ values of <2.5 µg·cm^−2^), an insect that causes great economic loss by destroying a variety of vegetables. The authors also reported the isolation of six new grayanane diterpenoids (**44**–**49**) (Figure 22) from *Pieris japonica* showing an unusual 3,4-*seco* A ring moiety. However, these new compounds showed weaker activity than grayanane diterpenoids with usual skeleton, suggesting that the integrity of the A ring was possibly vital for the maintenance of potent activity [103]. According to the authors, the analysis of structure-activity relationship indicated that the introduction of 2,3-expoxy moiety in the A ring, such as compound **43**, and the substitution of α-OH at the C-6 position in the B ring, such as compound **39**, can enhance the activity [103]. 

**Figure 23:**
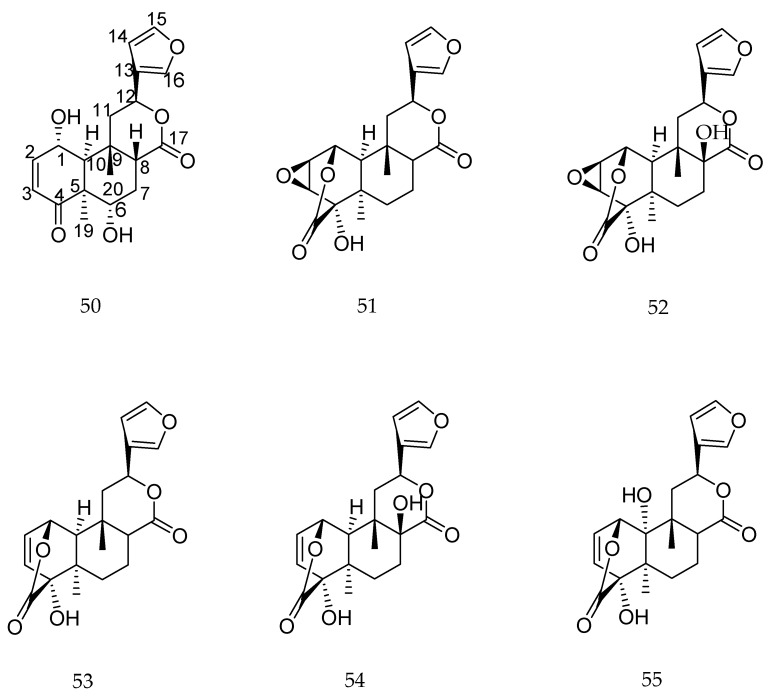
Chemical structures of diterpenes **50**–**55** [105].

The most studied biological property of clerodane-type diterpenes is the insect antifeedant activity [104]. Clerodane diterpenes such as tincordin (**50**), tinosporide (**51**), 8-hydroxytinosporide (**52**), columbin (**53**), 8-hydroxycolumbin (**54**), and 10-hydroxycolumbin (**55**) (Figure 23) were evaluated for their efficacy as insect antifeedants against *Earias vittella*, *Plutella xylostella*, and *Spodoptera litura* [105]. All diterpenes showed antifeedant activity and compound **52** was the most effective one [105]. 

Regarding the antifeedant activity of clerodane-type diterpenes, some authors have been tried to establish structure–activity relationships [106,107]. However, due to the absence of information on the modes of actions of these compounds and the biological targets involved, a few statements were established about structure–activity relationship as follow: (i) many active clerodanes possess the trans-decalin skeleton of the neo-clerodanes; (ii) the most active clerodanes have an oxygenated ring system at the sidechain-fragment at C-9; (iii) structural elements mentioned in the items i and ii must be present simultaneously; and (iv) structural elements mentioned in the items i and ii must be able to adopt a distinct special orientation [107]. 

**Figure 24:**
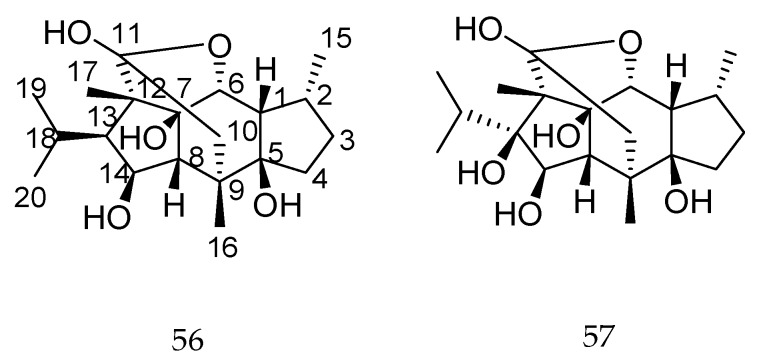
Chemical structures of diterpenes **56** and **57** [108].

Besides clerodane-type diterpenes, Tang et al. [108] reported that isoryanodane-type diterpenes (compounds **56** and **57**, Figure 24) showed antifeedant activity against various insect pests, and, according to the authors, the hydroxyl group at the C-14 position play an important role in their antifeedant activity. 

**Figure 25:**
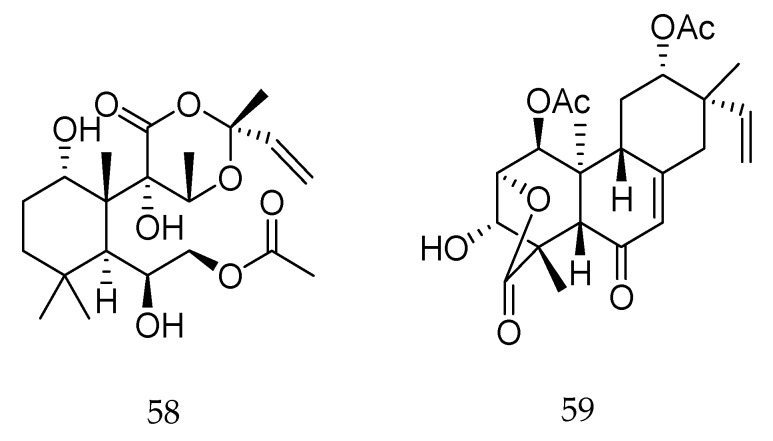
Chemical structures of diterpenes **58** and **59** [109,110].

Forskolin (**58**, Figure 25), a labdane-type diterpene, was reported by Vattikonda and Sangam [109] due to its destructive effects on *Papilio demoleus*, an insect that is a serious pest on *Citrus* sp. [109]. According to the authors, compound **58** induced morphological deformities and caused sterility of adults, suggesting its use as an insect growth regulator. 

Chenopodolin (**59**, Figure 25), a diterpene produced by the fungus *Phoma chenopodicola*, belongs to the group of tricyclic and tetracyclic unrearranged pimarane diterpenes. This compound caused the appearance of circular necrotic lesions when applied to detached leaves of *Mercurialis annua*, *Cirsium arvense*, and *Setaria viride* [110], suggesting its potential to be used as a natural and safe herbicide. 

**Figure 26:**
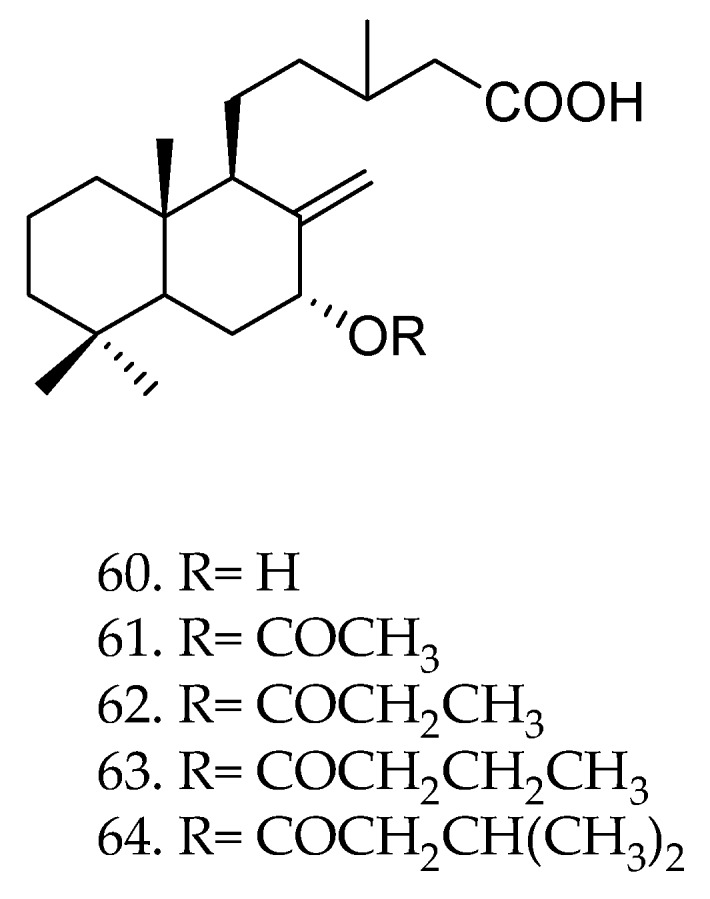
Chemical structures of diterpenes **60**–**64** [111].

Diterpenes from different sources have also been shown antifungal activity against a significant number of fungi including *Botrytis cinerea*, a phytopathogenic fungus that affects fruits, leaves, stems and flowers of more than 250 plant species [63,111]. The diterpenoids salvic acid (**60**, Figure 26), acetylsalvic acid (**61**), propanoylsalvic acid (**62**), butanoylsalvic acid (**63**), and isopentanoylsalvic acid (**64**) were evaluated against *Botrytis cinerea*. Compounds **60**, **61**, and **62** were the most active showing median effective doses (ED_50_) of 53.1 ± 4.6, 60.2 ± 8.4, and 59.5 ± 5.5 µg·mL^−1^, respectively [110]. According to the authors, diterpenoids with small and lipophilic groups were more effective than the derivatives with the longest chains [111]. 

**Figure 27:**
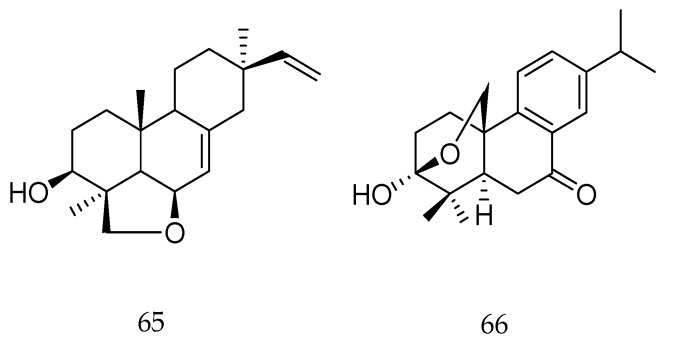
Chemical structures of diterpenes **65** and **66** [112].

A new pimarane diterpene, 6β,19β-epoxy-3β-hydroxy-5α,9β-pimara-7,15-diene (**65**, Figure 27), and a new abietane diterpene, 3β,20β-epoxy-3α-hydroxy-5α-abieta-8,11,13-trien-7-one (**66**), isolated from *Oryza sativa*, were evaluated against the fungus *Magnaporthe grisea* (causal agent of rice blast disease). Both compounds showed inhibitory activity against spore germination of the fungus with IC_50_ values of 0.213 and 0.232 mM [112]. 

Considering the potential of diterpenes to be used as insect antifeedant, insecticidal, and antifungal agrochemicals, and that synthetic agrochemicals are being discouraged because of the human health and environmental concerns [113], diterpenes appear to be an alternative for the development of new agrochemicals with improved selectivity without incurring the ecological risks associated with synthetic compounds. 

## 4. Conclusions

The biotransformation of diterpenes has proved to be an important tool to reach specific reactions and functionalization of deactivated carbons. From results gathered in this review, it should be highlighted that, for the first time, research reported an enzymatic Diels-Alder type reaction with a secondary metabolite in biotransformation studies and that micro-organisms were able to catalyze reactions of conjugation with amino acids, epimerization, and migration of the double bond, besides a rare example of diterpene halogenation. In addition, phase I and phase II diterpene metabolites were produced by animal and human liver microsomes and rats in vivo, providing better understanding of the active diterpenes metabolism. Finally, the knowledge of both the biological activities of diterpenes and the role of these compounds in nature may inspire the development of new agrochemicals with improved selectivity. 

## Figures and Tables

**Table 1:** Effects of biotransformation on the biological activities of different diterpenes.

Parent Compound	Biotransformation Biocatalyst	Biological Activity Evaluated	Enzymatic Reaction	Effect on Biological Activity *	References
sclareolide	fungi	cytotoxicity in vitro	hydroxylation	enhance	[72]
ingenol-3-angelate	plant cell cultures	cytotoxicity in vitro	Hydroxylation deacylation	decrease decrease	[66]
*ent*-15*α*-hydroxy-kaur-16-en-19-oic	fungi	allelopathic	hydroxylation	enhance	[73]
*ent*-8(14),15-pimaradiene	fungi	antibacterial	hydroxylation dihydroxylation	enhance no effect	[74]
mulin-11,13-dien-20 oic acid	fungi	gastroprotective in vivo	hydroxylation dihydroxylation	enhance enhance	[75]
20-deoxyingenol	fungi	cytotoxicity in vitro	hydroxylation	no effect	[81]
13-oxyingenol dodecanoate	fungi	cytotoxicity in vitro	hydroxylation	decrease	[81]
pseudolaric acid B	fungi	antifungal	conjugation with amino acids epimerization migration of double bond	decrease decrease decrease	[46]
*ent*-trachyloban-18-oic acid	fungi	cytotoxicity in vitro	hydroxylation backbone rearrangement	decrease decrease	[80]
(+)-(4*R*,5*S*,8*R*,9*S*)-18-hydroxy-*ent*-halima-1(10),13-(*E*)-dien-15-oic	fungi	anticholinesterase (Hr-AChE)	oxidation carboxylation hydroxylation	enhance enhance enhance	[55]
cryptotanshinone	fungi	antiviral	Degradation and rearrangement	enhance	[54]
16-oxacleroda-3,13(14)*E*-dien-15-oic acid	fungi	antifungal	hydroxylation	enhance	[82]
trachyloban-19-oic acid	fungi	anticholinesterase	oxidation rearrangement	enhance enhance	[65]
10-oxo-2*R*,5*R*,14β-triacetox-ytaxa-4(20),11(12)-diene	bacteria	tumor MDR reversal activity	hydroxylation dihydroxylation oxidation and acetalization	no effect no effect enhance	[59]
deoxyandrographolide	fungi	LPS-induced NO production	oxidation of –OH to ketone hydroxylation epoxidation cleavage of the lactone	enhance decrease decrease decrease	[77]
andrographolide	fungi	cytotoxicity in vitro	oxidation of –OH to ketone hydration epimerization	decrease decrease enhance	[56,57]

* When compared to the parent compound.
